# Fun

**Published:** 1859-10

**Authors:** 


Fun is worth more than physic, and whoever invents or discovers a new source of supply, deserves the name of a public benefactor; and whoever can write an article the most laughter-promoting and at the same time harmless, is worthy of our gratitude and respect, both of which we hold subject to the order of the writer for the *New York Evening Post* of the following:

"On the afternooon of the fourth of July there was a wheelbarrow race at the Phalanx, New Jersey, the men taking part therein being blindfolded. The ground in front of the hotel slopes to a small lake, and has a number of trees. The wheelbarrows were placed upon the border of the water at the foot of the slope, and turned directly away from the point to which the contestants were to go. The eyes of those who entered the lists were then bandaged, and the master of ceremonies gave the words: 'Ready! take hold of your wheelbarrows! about face? forward!'

"A barrel was placed in the drive before the hotel, and the man whose vehicle should be found nearest this point when the drive in front of the house was reached was to gain the victory. Before being blindfolded, of course, each contestant took a fond, farewell look at the barrel, endeavoring to fix the direction firmly in his mind. But when the word 'about face' was obeyed, most of them lost it; some struck off in one direction and some in another, to the great merriment of the spectators, who witnessed the important contest from the balcony of the hotel. On one hand a blind mortal made an angle of forty-five degrees to the east, while on the other an equally blind mortal made an equally ludicrous blunder in the opposite direction; and in the centre the sightless array staggered on like a cohort of discouraged soldiers under a heavy fire. Now one of them encounters a tree in his blind march, and is brought to a dead stand still. The shock not only produces an unpleasant sensation in his arms, but quite confuses his few remaining notions of the points of compass. He hesitates, turns in all directions to satisfy himself, and finally strikes off on a course nearly opposite the point to be gained. Now, again, a man steps upon the root of a tree, and loses his footing and sense of direction together; and now, in their confused ramblings, two straggling competitors meet each other and get their wheelbarrows locked together. This convinces each that he was wrong; so both face about.

"One by one they reach the drive, some a few rods from the barrel, but most at a ludicrous distance. One man finds himself still at the base of the slope, as far from the goal as when he was bandaged. Another has traveled three times the distance, and brought up at the extreme end of the drive. Another has passed over the gravel without knowing it, and finds himself in the field beyond.

"But there was one who had a decided advantage, being partially blind, and accustomed to trust his instincts in this manner. The bandage was a small inconvenience to him, and as soon as the word was given, he marched directly for the barrel, and deposited his wheelbarrow within a single pace of it, while the others were engaged in their serpentine travels."

